# Pediatric Suprasellar Germ Cell Tumors: A Clinical and Radiographic Review of Solitary vs. Bifocal Tumors and Its Therapeutic Implications

**DOI:** 10.3390/cancers12092621

**Published:** 2020-09-14

**Authors:** Darian R. Esfahani, Tord Alden, Arthur DiPatri, Guifa Xi, Stewart Goldman, Tadanori Tomita

**Affiliations:** 1 Division of Pediatric Neurosurgery, Ann & Robert H. Lurie Children’s Hospital, Chicago, IL 60611, USA; darian.esfahani@gmail.com (D.R.E.); talden@luriechildrens.org (T.A.); adipatri@luriechildrens.org (A.D.); gxi@luriechildrens.org (G.X.); 2 Department of Neurosurgery, Northwestern University Feinberg School of Medicine, Chicago, IL 60611, USA; 3 Division of Hematology, Oncology, Neuro-Oncology & Stem Cell Transplantation, Ann & Robert H. Lurie Children’s Hospital, Chicago, IL 60611, USA; sgoldman@luriechildrens.org

**Keywords:** intracranial germ cell tumor, germinoma, suprasellar tumor, dual germ cell tumor, bifocal germ cell tumor, non-germinoma germ cell tumor, magnetic resonance imaging, radiation, overall survival, progression free survival

## Abstract

**Simple Summary:**

Bifocal suprasellar germ cell tumors are a unique type of an uncommon brain tumor in children. Compared to other germ cell tumors in the brain, bifocal tumors are poorly understood and have a bad prognosis. In this paper we explore features that predict which children will have good outcomes and which will not. This is important for the research community because it can help physicians decide what type of radiation treatment is best to treat these children. Our study shows that bifocal tumors have a unique appearance on magnetic resonance imaging (MRI) compared to other germ cell tumors. Children with bifocal tumors are more likely to be male, have tumors that come back sooner, and cause death sooner. We found that children with bifocal tumors likely need a wider radiation field, especially if they have a high-risk tumor type, high-risk appearance on MRI, or tumors spread throughout the nervous system.

**Abstract:**

Suprasellar germ cell tumors (S-GCTs) are rare, presenting in either solitary or multifocal fashion. In this study, we retrospectively examine 22 solitary S-GCTs and 20 bifocal germ cell tumors (GCTs) over a 30-year period and demonstrate clinical, radiographic, and prognostic differences between the two groups with therapeutic implications. Compared to S-GCTs, bifocal tumors were almost exclusively male, exhibited higher rate of metastasis, and had worse rates of progression free and overall survival trending toward significance. We also introduce a novel magnetic resonance (MR) imaging classification of suprasellar GCT into five types: a IIIrd ventricle floor tumor extending dorsally with or without an identifiable pituitary stalk (Type Ia, Ib), ventrally (Type III), in both directions (Type II), small lesions at the IIIrd ventricle floor extending to the stalk (Type IV), and tumor localized in the stalk (Type V). S-GCTs almost uniformly presented as Type I–III, while most bifocal GCTs were Type IV with a larger pineal mass. These differences are significant as bifocal GCTs representing concurrent primaries or subependymal extension may be treated with whole ventricle radiation, while cerebrospinal fluid (CSF)-borne metastases warrant craniospinal irradiation (CSI). Although further study is necessary, we recommend CSI for bifocal GCTs exhibiting high-risk features such as metastasis or non-germinomatous germ cell tumor histology.

## 1. Introduction

Intracranial germ cell tumors (GCTs) are an uncommon malignancy that represent approximately 0.9% of all pediatric tumors, 3.7% of pediatric brain tumors, and 28.7% of germ cell tumors overall [1,2]. However, GCT presentation is very heterogenous, without uniform imaging classification. GCTs occur most often in the pineal (P-GCT) and suprasellar (S-GCT) locations, followed by the basal ganglia (BG-GCT) and rarely at other central nervous system (CNS) sites. GCTs can also arise concurrently in the suprasellar and pineal regions, a distinct pathologic entity described as “bifocal” GCTs [3,4,5,6]. Bifocal tumors are especially poorly understood, with few studies analyzing their natural history or comparing outcomes versus solitary GCTs. It is further unclear whether bifocal GCTs represent independent primaries or metastases, with the difference significant for treatment. Independent primaries, for example, are suitable for limited radiation fields, while and metastases warrant full craniospinal irradiation.

Per the Central Brain Tumor Registry of the United States, GCTs represent 3.9% of brain tumor patients under the age of 20 [7]. Racial differences exist, with some registries reporting a higher overall incidence of germ cell tumors in patients of Asian descent. The frequency of GCTs in Japan was 16.9% among brain tumor patients under the age of 20, making them the second most common brain tumor type after astrocytoma [8]. According to a Children’s Oncology Group report, the tumor location of 93 intracranial GCTs were P-GCT in 53%, S-GCT in 37%, and bifocal in 5.4%, while a Japanese cooperative study demonstrated P-GCT in 68%, 26 S-GCT in 19%, and bifocal GCT in 13% among 139 GCTs [8]. A comparison study between the Mayo Clinic and Japan National Cancer Center (NCC) demonstrated the frequency of basal ganglia GCTs was higher in the NCC database compared with the Mayo Clinic (8.4% vs. 0%), and bifocal location (S-GCT and P-GCT) was higher at the Mayo Clinic than at the NCC (18.8% vs. 5.8%) [9].

Many authors consider GCTs to be derived from the abnormal migration of germ cells along the midline during embryonal development, where they present along a longitudinal axis from the optic chiasm to the pineal region in the III ventricle. Among GCTs, germinomas of the pineal and suprasellar region are the most common, followed by tumors along the ventricle [3,9,10,11]. It has been considered that P-GCTs are derived from ectopic cells that migrate to the pineal region and S-GCTs derive from ectopic germ cells that migrate to the hypothalamic infundibulum or neurohypophysis. The pathogenesis of bifocal GCTs is unclear as to whether bifocal GCTs represent tumors developing at both sites simultaneously or are metastases from one site to the other. Takami et al. reported the majority of bifocal or other multifocal tumors (34/35 cases, 97.1%) were germinomas [8]. Literature showing a high rate of tumor seeding (47.8%) in bifocal GCTs and a worse survival in bifocal GCTs supports the metastasis hypothesis [5,6]. Based on clinical and imaging characteristics, Zhang et al. recently suggested a distinction between suprasellar tumors of “true” bifocal GCTs that arise from the neurohypophysis and those of “false” lesions that are metastatic [12]. If the primary lesions of bifocal GCTs are suprasellar, one would suspect they should share similar magnetic resonance (MR) imaging features and initial clinical symptoms as S-GCTs [13].

Currently, there is no consensus of radiation therapy (RT) field for bifocal GCTs. Implicit in treatment choice is the definition of what a bifocal GCT is: if a bifocal GCT represents disseminated disease, it should be treated with full craniospinal irradiation (CSI); if it represents localized disease, treatment with limited fields is more appropriate. This distinction is important for GCT treatment using radiation therapy (RT), as metastatic GCTs likely warrant full craniospinal irradiation (CSI), while localized lesions require only limited field irradiation, which carries less morbidity to the developing nervous system of children [6,14,15,16,17].

To better characterize the clinical and radiographic presentation of bifocal GCTs and identify whether they represent independent primaries or metastases, in this study, we retrospectively analyze a population of GCTs, focusing on their clinical presentation, imaging characteristics, and responses to the therapy.

## 2. Results

### 2.1. Clinical Presentation

Eighty-four patients with intracranial GCT were diagnosed and treated during the 30-year study period and retrospectively analyzed. Of these, 42 were solitary pineal GCTs, while the remaining 42 involved the suprasellar region. Of the suprasellar tumors, 22 were confined to the suprasellar region (S-GCT), 16 were also present in the pineal region (bifocal GCT) and four were also present in the basal ganglia (BG-GCT). Among the 42 GCTs involving the suprasellar location, six were managed from 1988 to 1999, and the remaining 36 were managed within the last 20 years.

Patient age at diagnosis ranged from six to 18 years of age (mean: 12 years). Children with S-GCT were significantly younger (11.2 years) versus children with bifocal GCTs (13.4 years; *p* = 0.04). There were 16 females and 26 males. Children in the S-GCT group were much more likely to be female (68.2%), while children in the bifocal GCT group were all male except for one (6.3%) (*p* < 0.001), with an odds ratio (OR) of 32.71 (confidence interval [CI] 3.51-294.23).

The most common presenting symptom was diabetes insipidus (DI). DI was present in all but one S-GCT patient (95.5%), and in 12 of 16 (75%) and three of four (75%) of the bifocal GCT and BG-GCT groups, respectively. The duration of DI before tumor diagnosis ranged from 0 (at the time of tumor detection) to three years; 13 patients had DI for more than one year of duration (median average; 14.5 months among S-GCT, 7.3 months bifocal, and 2.3 months for BG-GCT).

Other endocrine dysfunctions were also common at patient presentation. Among the 22 patients with S-GCT, short stature was present in five (22.7%), delayed puberty in three (13.6%), precocious puberty in two (9.1%), excess body weight gain in five (22.7%), and weight loss in two (9.1%). Among the bifocal GCT patients, three (18.8%) had excess weight loss prior to diagnosis. Two patients in the BG-GCT group had endocrine dysfunction, with precocious puberty in one and thyroid and adrenal insufficiency in the other.

Visual symptoms were the next most common presenting symptom and were present in nine (40.9%) of the S-GCT and five (31.3%) of the bifocal patients. Three patients (75%) with BG-GCT presented with hemiparesis or involuntary motor movement and then developed DI subsequently. Hydrocephalus was common in the bifocal group and present in 10 (62.5%) of patients, a significantly greater rate than in the S-GCT group (3/22, 13.6%; *p* = 0.019) with an OR of 10.56 (2.17–51.43). No instances of hydrocephalus were observed in the BG-GCT group.

### 2.2. Tumor Markers

Tumor marker data was available in 36 (85.7%) patients. Fifteen (41.7%) were negative for both markers. Serum alpha fetoprotein (AFP) was elevated in six of 20 (30%) tested patients and ranged from 11.9 to 8,000 ng/mL. Cerebrospinal fluid (CSF) AFP was elevated in four of nine (44.4%) patients and ranged from 2.5 ng/mL to 240 ng/mL.

Among 36 patients tested for both CSF and serum beta human chrorionic gonadotropin (β-HCG), 21 (58.3%) demonstrated abnormal values. Serum β-HCG was positive in only eight patients (22.2%) and ranged from 8 IU/L to 312 IU/L. In all but one patient, higher β-HCG titers were noted in the CSF than serum. The range of the β-HCG values in the CSF was 5–10 IU/L in eight patients, 11–50 IU/L in seven patients, and 51–100 IU/L in two patients. The remaining four patients had CSF values higher than 100 IU/L; 120, 131, 341, and 984 IU/L, respectively.

### 2.3. MR Imaging

MR characteristics of suprasellar masses were classified by location and involvement of the pituitary stalk (PS) and pituitary gland (PG) and are described in detail in the Materials and Methods section. A summary by imaging type and location is demonstrated in Table 1. The size of the sella turcica was normal in size in all except for three patients of Type II and III, where a mildly enlarged sella turcica was noted. All GCTs in every group demonstrated contrast enhancement.

**Figure 1:**
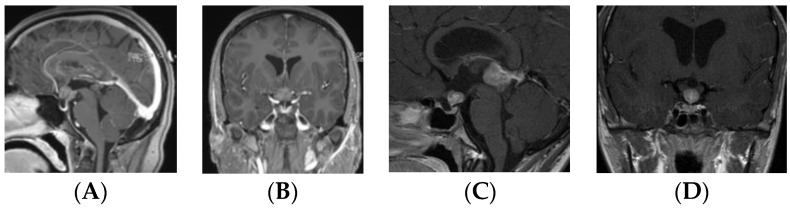
Type Ia. Post-contrast MR, sagittal (**A**) and coronal (**B**) views of a patient with suprasellar germ cell tumor (S-GCT) and post-contrast MR, sagittal (**C**) and coronal (**D**) views of a patient with bifocal GCT. Note an anterior third ventricle tumor with preserved pituitary stalk and gland.

**Figure 2:**
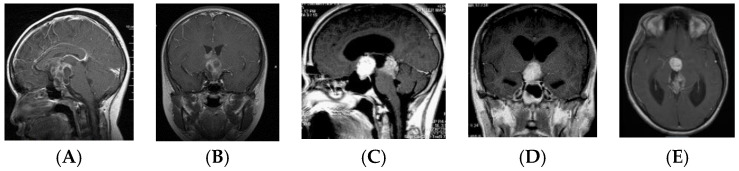
Type Ib. Post-contrast MR, sagittal (**A**) and coronal (**B**) views of a patient with S-GCT and post-contrast MR, sagittal (**C**), coronal (**D**) and axial (**E**) views of a patient with bifocal GCT. The pituitary stalk is not visualized but the pituitary gland is preserved. Note the subependymal infiltrate of at the III ventricle wall (**E**).

**Figure 3:**
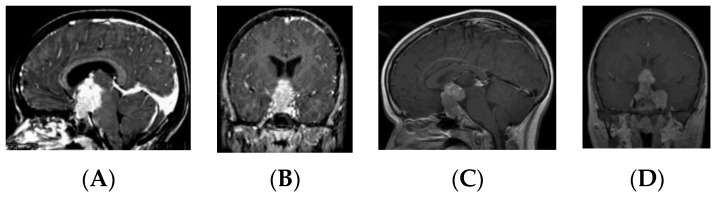
Type II. Post-contrast MR, sagittal (**A**) and coronal (**B**) views of a patient with S-GCT. Note the third ventricle tumor extension into the sella turcica. Post-contrast MR, sagittal (**C**) and coronal (**D**) views of a patient with S-GCT demonstrate further lateral extension to the cavernous sinus.

**Figure 4:**
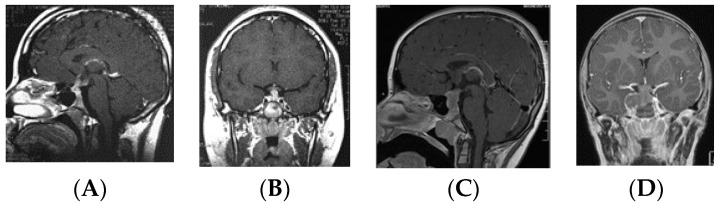
Type III. Post-contrast MR, sagittal (**A**) and coronal (**B**) views of a patient with S-GCT. Note the tumor from the floor of the third ventricle to the sella turcica. Post-contrast MR, sagittal (**C**) and coronal (**D**) views of a patient with S-GCT. Note the tumor from the floor of the third ventricle to sella turcica with further extension into the cavernous sinus.

Patients in the S-GCT group skewed toward masses of the anterior III ventricle. Of 22 S-GCTs: seven were Type Ia or Ib (Figure 1 and Figure 2), seven were Type II (Figure 3), and six were Type III (Figure 4). These 20 S-GCTs were all globular appearance. In four patients (two each of S-GCT Type II and III), the tumor extended laterally into the cavernous sinus (Figure 3C,D; Figure 4C,D). Each of these patients presented with cranial nerve dysfunction. The two non-globular S-GCTs were small and confined to the PS (Types IV and V).

**Figure 5:**
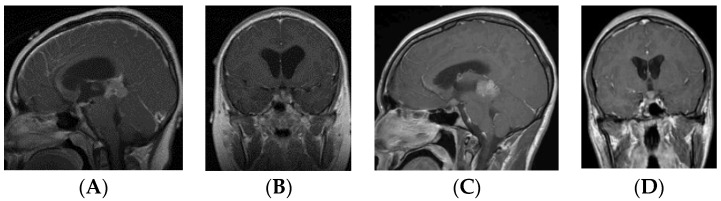
Type IV. Post-contrast MR, sagittal (**A**) and coronal (**B**) views of a patient with bifocal germ cell tumor (GCT). Note a laminar tumor at the floor of the third ventricle with upper pituitary stalk involvement. Post-contrast MR, sagittal (**C**) and coronal (**D**) views of a patient with bifocal GCT with a nodular tumor at the floor of the third ventricle with upper pituitary stalk involvement.

By contrast, the majority (11/16, 68.8%) of bifocal GCTs were smaller and Type IV (Figure 5). These tumors were accompanied by much larger pineal region tumors. Four bifocal tumors were Type Ia or Type Ib tumors with similarly sized globular masses in the suprasellar and pineal regions. 1 patient had a Type II mass with extension of the tumor directly into the sella turcica.

**Figure 6:**
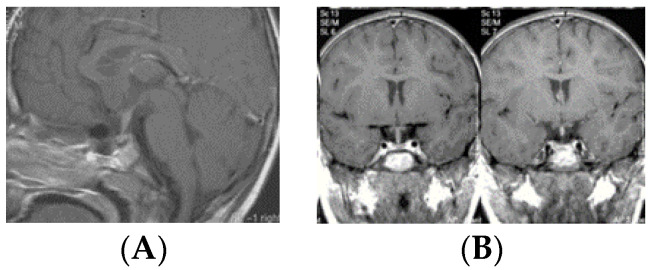
Type V. Post-contrast MR, sagittal (**A**) and coronal (**B**) views of a patient with S-GCT. Note the thickened pituitary stalk. This seven-year-old girl presented with 18-month history of DI.

There were only three Type V tumors (Figure 6), and two of these were BG-GCTs. All four BG-GCTs demonstrated a prominent PS. Two of these had poorly defined hyperintense lesions extending from the BG to corpus callosum, but a prominent PS was present. The other two BG-GCTs were Type III and were more extensive enhancing lesions in the basal ganglia extending through the anterior cingulate gyrus.

Metastases were more common among the bifocal GCT (7/16) and BG-GCT (3/4) groups versus the S-GCT group (4/22), with the combined bifocal and BG GCT patient population significantly more likely to have metastases than their S-GCT counterparts (*p* = 0.049) with an OR of 4.5 (CI: 1.12i18.13). All metastases at diagnosis were noted in the lateral ventricle, except for one in the fourth ventricle and another in the subarachnoid space over the cerebellum. There were no spinal metastases at the time of initial diagnosis on spinal MR.

**Figure 7:**
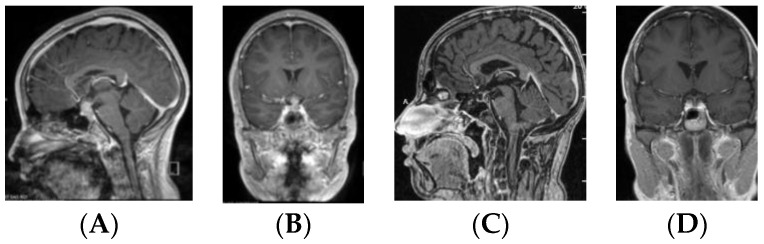
Thirteen-year-old male with suspected germinoma with high cerebrospinal fluid (CSF) β-HCG presenting with a one-month history of DI and monocular visual loss. Post-contrast MR, sagittal (**A**) and coronal (**B**) views showing an enhancing mass with Type II suprasellar lesion. Following pre-RT chemotherapy, the lesion resolved on postcontrast MR sagittal (**C**) and coronal (**D**) views. Note normal continuity of the pituitary stalk and atrophic pituitary gland.

**Figure 8:**
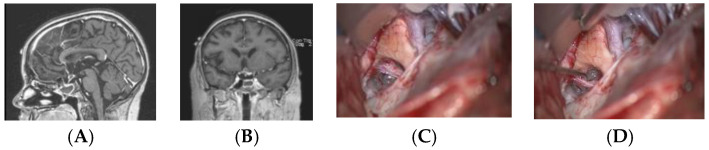
Twelve-year-old girl with suprasellar tumor with β-HCG. Following chemotherapy, the β-HCG was normalized, but there was a small contrast-enhancing lesion in the left suprasellar location on MR (sagittal (**A**) and coronal (**B**) views). Surgical photographs at second-look surgery demonstrate the chiasm and an atrophic but continuous pituitary stalk (**C**), which is retracted by a hook (**D**). The lesion demonstrated no viable cells.

Except for the four patients who underwent primary surgical resection, MR imaging following adjuvant therapy demonstrated lesion resolution in all patients with a preserved PS and either a normal or atrophic PG (Figure 7). The PS further demonstrated anatomic continuity during second-look surgery (Figure 8).

### 2.4. Surgery

Of 42 patients, 28 (66.7%) underwent surgical resection or biopsy, 11 underwent a trans-ventricular endoscopic approach, nine an open craniotomy, five a transsphenoidal approach, and three a stereotactic biopsy (Table 2). No differences were noted between the propensity of different GCT types to undergo surgery. Expectedly, bifocal GCTs were more likely to undergo endoscopic biopsy than S-GCTs (*p* = 0.003), while S-GCTs were more likely to undergo craniotomy (*p* = 0.012). The remaining 14 patients had positive tumor markers and were treated with upfront chemotherapy without surgical biopsy. Three of these patients subsequently underwent second-look surgery by craniotomy, and one by the transsphenoidal approach.

### 2.5. Pathology

Pathology results are illustrated in Table 3. Diagnosed (24) and suspected (nine) germinomas were the most common in the series, constituting 33 of 42 (78.6%) of this cohort. All suspected germinomas had elevated β-HCG in the CSF and demonstrated a complete response to neoadjuvant chemotherapy. Non-germinomatous germ cell tumors (NGGCTs) were most common in the S-GCT group (7/22, 31.8%), were uncommon in the bifocal group (2/16, 12.5%), and absent among BG-GCTs, although these differences did not reach significance. Of the nine NGGCTs, five were AFP producing and three had high β-HCG, which set at a threshold of greater than 100 IU/L. One patient had both high AFP (222 ng/mL in serum and 90 ng/mL in CSF) and β-HCG (312.4 IU/L in serum and 984 IU/L in CSF). While almost all patients with a β-HCG greater than 100 IU/L were NGGCTs, this was not universal, with a single biopsy-confirmed germinoma having a CSF level of 180 IU/L.

### 2.6. Adjuvant Therapy

Neoadjuvant chemotherapy prior to RT was applied to all but one patient. Most patients were enrolled in Children’s Oncology Group (COG) germ cell protocol ACNS0122, 0232, and most recently in ACNS 1123. 1 patient had on COG ACNS0232 randomization, and received only RT.

All patients subsequently received RT. Radiation fields are described in Table 4. Whole ventricle (WV) with focal boost was applied to 17 patients, all with germinomas and three with evidence of intra-ventricular metastases. Craniospinal irradiation (CSI) with tumor bed boost was applied to 19 patients including seven NGGCTs; among them, 10 demonstrated intraventricular metastases. Intraventricular metastases were significantly more common among patients undergoing CSI (52.6%) than those undergoing WV therapy (17.6%; *p* = 0.041), with an OR of 5.19 (1.11–24.14). The remaining five patients had limited field radiation with intensity modulated RT. 1 patient treated very early in the study had whole brain (WB) radiation.

### 2.7. Clinical Outcomesq456

All patients had initial response to pre-RT chemotherapy with total or near total resolution except for four patients with NGGCT who underwent a second look surgery for resection of a residual lesion. The second look surgery demonstrated a mature teratoma in two S-GCT patients, one of which was removed by craniotomy and another by a transsphenoidal approach. The other two patients demonstrated only scar tissue without viable tumors.

Progression free and overall survival were the two clinical outcomes analyzed in this study. Children in the S-GCT, bifocal, and BG-GCT groups were followed for a mean of 9.5, 6.6, and 5.7 years, respectively. Among patients who survived until the end of the study period, the suprasellar group was followed for a mean of 9.0 years, the bifocal group for 7.8 years, and the BG-GCT group for 5.7 years, a difference that was not significant (*p* = 0.23).

**Figure 9:**
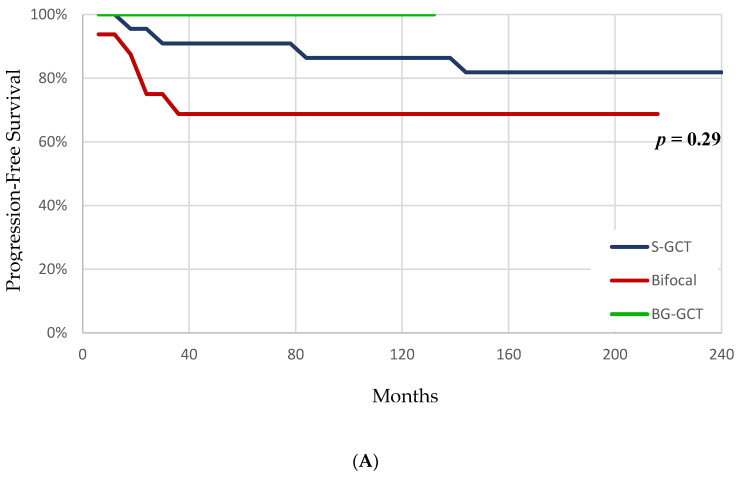

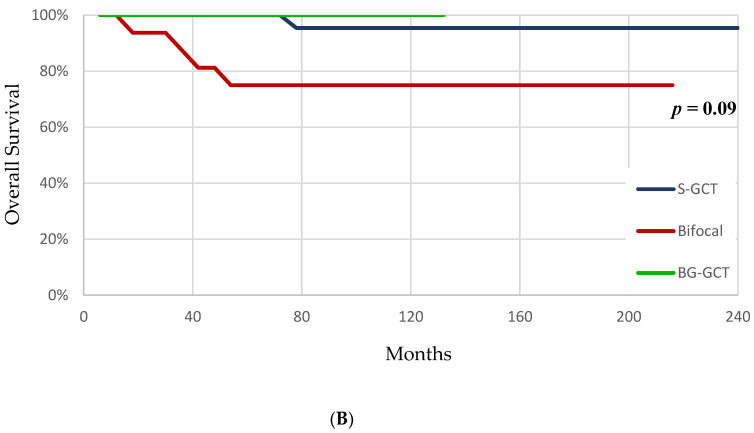
Progression free and overall survival. Kaplan–Meier survival curves illustrating progression free survival (**A**) and overall survival (**B**) for patients with S-GCT, bifocal, and basal ganglia (BG)-GCT. Children with bifocal tumors trended toward worse overall survival versus the other groups (*p* = 0.09).

Kaplan–Meier plots for progression free and overall survival are illustrated in Figure 9. At the end of the study period, 18/22 (81.8%) patients in the S-GCT group, 11/16 (68.8%) in the bifocal, and 4/4 (100%) of the BG-GCT group did not demonstrate tumor recurrence or progression, although this did not achieve significance (*p* = 0.29). (Figure 9A). By the end of the study period, 21/22 (95.4%) patients in the suprasellar group survived, versus 12/16 (75%) in the bifocal and 4/4 (100%) in the BG-GCT groups, a trend toward significance (*p* = 0.09) (Figure 9B).

Table 5 illustrates the relationships between tumor histology, applied radiation fields, and tumor recurrence. Nine patients had recurrence during the follow-up period: four S-GCTs, five bifocal GCTs, and no BG-GCTs.

Germinomas had excellent responses to chemo-radiotherapy with a recurrence rate around 10%, whereas NGGCT were much higher, at 50%. Of the four S-GCTs that recurred, three were NGGCT and one was a germinoma. The latter showed a single spinal metastasis two years after WV irradiation. One patient with NGGCT with high AFP and β-HCG developed recurrence 11 years later with an intrasellar mature teratoma. Another patient with NGGCT (high β-HCG) developed suprasellar and subarachnoid dissemination 18 months after limited field RT; she died of tumor six years after the diagnosis. The other patient with NGGCT (high AFP) had a local recurrence seven years after CSI RT with a histologically confirmed germinoma. Five bifocal GCTs recurred: three were germinomas and two were NGGCTs. Three were treated with CSI RT, and two germinomas received WV RT.

## 3. Discussion

### 3.1. Pathogenesis of Intracranial GCT

The pathogenesis of intracranial GCTs remains poorly understood. Teilum proposed that germ cell progenitors mis-migrate during early embryogenesis, become trapped in the midline axis, and become a source of GCTs [18]. The mechanism of this mis-migration remains unknown. All GCTs develop from a common precursor, a totipotent primordial germ cell capable of embryonic and extraembryonic differentiation irrespective of site [19]. However, Sano theorized that the only “true” GCT is the germinoma, while the others, NGGCT, are not from the primordial germ cell but should be regarded as enfolded-cell-derived dysembryogenetic cells [20].

The presence of GCT in specific diencephalic loci suggests that local factors are important in either attracting germ cells to these sites or altering normal patterns of development [21]. Jennings et al. theorized that the neuroendocrine events of puberty are an “activating” influence in the malignant expression of embryonal tumors due to their specificity of origin within the positive (suprasellar cistern–suprachiasmatic nucleus) and negative (pineal) regulatory centers for gonadotropin secretion within the diencephalon [21].

Stem-cell-related proteins (C-KIT, OCT-3/4 (POU5F1), AP-2γ (TFAP2C), and NANOG) and developmentally regulated germ-cell-specific proteins are highly expressed in CNS GCTs [22]. The expression of genes associated with embryonic stem cell pluripotency indicates that CNS GCTs are derived from cells that retain, at least partially, an embryonic stem-cell-like phenotype, which is a hallmark of primordial germ cells [22]. These GCTs arise from the transformation of endogenous brain cells and likely originate from neural progenitors rather than mis-migrated germ cell progenitors. Neural stem cells acquire or maintain OCT4 expression to form a cell with pluripotent features. Gene activation of OCT4 by demethylation, together with the local high hormonal activity such as IGFs and GnRH, may be important in the transformation of cells in the ventral midline brain toward tumor formation [23].

### 3.2. Suprasellar GCT

Based on our MR classification, isolated S-GCTs predominately either arise from the III ventricle floor dorsally to the III ventricle cavity (Type I), from the floor ventrally to the suprasellar/sellar location (Type III) or both directions (Type II). A minority are present at the median eminence to the upper PS (Type IV) or only at the PS (Type V). None of our patients had a localized GCT within the sella turcica at the neurohypophysis at tumor diagnosis. Based on this observation, it appears that the tuber cinereum and the median eminence through the pituitary stalk are most likely the site for the origin of S-GCTs.

Nearly all S-GCTs present with DI, which may be latent and last for several years, as demonstrated in our series and others [12,23]. The presence of DI among intracranial GCTs strongly indicates suprasellar disease, and endoscopic visualization is often more sensitive to confirm this than MR findings [24]. In addition, patients with S-GCT often present with other endocrine disorders indicative of hypothalamic dysfunction such as excess changes in body weight and precocious or delayed puberty. Of interest, following successful therapy, the pituitary stalk was noted to be in anatomic continuity from the floor of the III ventricle to the normal or atrophic pituitary gland in each group. It is a common finding that the PS reappears or normalizes after treatment even if not initially visualized or enlarged from tumor involvement on pre-treatment MR. This finding suggests GCT cells infiltrate the stalk rather than replace cells of the hypothalamoneurohypophyseal axis (HNA).

#### 3.2.1. Bifocal Germ Cell Tumors

Bifocal intracranial GCTs are a distinct, unique presentation of intracranial germ cell tumors that, while described in the literature, remain poorly understood [3,4,5,6]. Attributable to their dual nature, the epidemiology of bifocal GCTs carries features common to both suprasellar and pineal region GCTs, a finding also exhibited in our series. The incidence of bifocal GCTs are reported 5–25% among intracranial GCTs [4,6,11].

#### 3.2.2. Demographics

The mean age of bifocal tumors in this cohort was 13.3 years of age, significantly older than the suprasellar group. While the overall cohort age was similar to pediatric databases and studies [1,5], the reason the bifocal GCT group skewed older is not clear. Most of bifocal GCTs are germinomas which tend to present at an older age than the other histology groups [8]. Another hypothesis is that the bifocal group may represent a more advanced form of the disease, with the additional time allowing for either the tumor cells to propagate from one site to the second or new, spontaneous lesions to arise [5,12].

Significant sex differences exist among intracranial GCTs, which are anywhere from 1.8 to 3.3 times more prevalent among males than females [1,25]. This difference varies by location, however, with a recent SEER registry study demonstrating sellar region GCTs proportionally half as common among males (13%) versus females (25%), while pineal region GCTs are markedly more frequent in males (61%) than females (16%) [25]. Other registry studies have found similar findings, with GCTs of the pineal gland 14–21 times more common among males than females, while the ratio was much lower for GCTs located elsewhere [9,26]. This study supports these earlier findings, with the suprasellar group 68% female and the bifocal GCT group almost exclusively male.

The finding that the bifocal GCT group exhibited a sex distribution pattern closer to the pineal group is potentially revealing; this supports that bifocal GCTs either arise originally from pineal masses that spread to the suprasellar region or, conversely, rarely occur in females. One mechanism for the rarity of pineal GCTs in females is that GCTs are specific to the positive (suprasellar hypothalamic nucleus) and negative (pineal) regulatory centers for gonadotropin secretion within the diencephalon [21]. Regardless, this finding suggests that if bifocal tumors represent the spread of the GCTs from one area to the next, it most often occurs from the pineal region to the suprasellar region, and not the reverse. If suprasellar GCTs were to spread to the pineal gland, a much greater proportion of female patients in the bifocal group would be expected.

#### 3.2.3. Symptoms

Among the bifocal GCT group, 12 (75%) patients initially presented with DI. Of 5 Type I and II bifocal GCTs, all had DI, with one at presentation and four within 18 months of diagnosis. Of the 11 patients with Type IV bifocal GCTs, seven (64%) presented with DI, with three at the time of diagnosis, two within one month, and two within three years. Bifocal GCTs of the patients who presented with early DI, neuroendocrine dysfunction, or visual impairment are suspicious for primary suprasellar lesions rather than secondary. Conversely, those that presented with hydrocephalus from a pineal mass or developed delayed DI are more likely to represent secondary suprasellar lesions. Thus, there are two distinct presentations among bifocal GCT, one potentially of suprasellar origin and another of pineal origin, based upon clinical symptomatology. Based on our MR classifications, 7/9 (77.8%) pineal-primary and 4/7 (57.1%) suprasellar-primary tumors were Type IV, and the rest were either Type I subtype.

#### 3.2.4. Pathomechanisms of Bifocality

Two potential mechanisms explain the pathophysiology of bifocal GCTs: 1) true multicentric synchronous occurrence in the suprasellar and pineal regions or 2) the spread from one site to the other.

The mechanism of GCT “spread” from one site to the other remains unclear. GCTs disseminate both by infiltration into the adjacent hypothalamus and via the ventricular and subarachnoid pathways [21]. If CSF-borne metastasis of cells is the mechanism, one would expect a higher incidence of lesions in the other ventricles or subarachnoid spaces. In our cohort, half (10/20) of multifocal GCTs had intraventricular seeding on MR with two positive cytology results. The preferential localization in the suprasellar or the pineal region by CSF-borne metastases is challenging to explain, but may represent relatively stagnant CSF flow within the III ventricle at the infundibular recess and pineal-suprapineal recess, as shown by Holmlund et al. [27]. We postulate stagnant CSF flow, compounded with vascularity at the median eminence and pineal gland as circumventricular organs may provide favorable nesting sites for GCTs. Another mechanism of GCT spread is direct subependymal extension along the floor or walls of the III ventricle. It has been suggested that the tumor develops either multicentrically or by subependymal laminar infiltration around the third ventricle when it occurs in multiple midline sites, rather than by subarachnoid or CSF pathway metastasis [28]. However, radiographic evidence of subependymal infiltration between the suprasellar and pineal lesions is rare on MR (Figure 2E).

**Figure 10:**
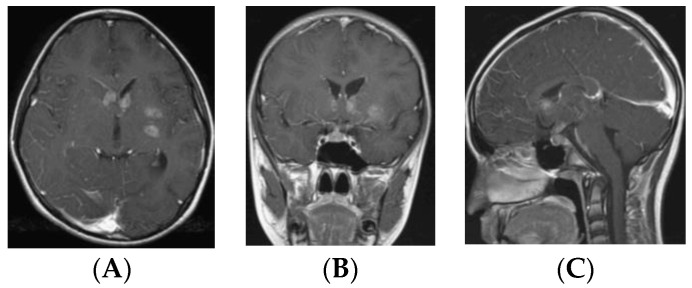
A 10-year-old boy with a month history of progressive right sided hemiparesis and headaches. On admission he was noted panhypopituitarism including DI. Post-contrast MR, axial (**A**), coronal (**B**), and sagittal (**C**) images showing para-ependymal enhancements with Type III suprasellar lesion.

Rarely, bifocality is noted with germinomas occurring in the suprasellar and basal ganglia [29]. In our cohort, the incidence of radiographic intraventricular disseminations was high among the BG-GCT group. However, there is a possibility of direct subependymal tumor extension along the third ventricle wall (hypothalamus) (Figure 10).

The relationship between bifocal GCT and ventricular metastases is supported by our study, with half of bifocal GCTs presenting with metastasis, four and a half times greater odds than lesions confined to the suprasellar region. Expectedly, the bifocal group trended toward worse overall survival (Figure 9b), likely for this reason. Previous literature has reported a similar rate of metastasis, with a paper by Weksberg et al. showing 25/55 (45%) patients among bifocal lesions exhibiting ventricular or CSF positive disease, similar to our rate of 50% among the multifocal GCT group [6]. Weksberg et al. further reported a worse five-year progression free survival (80%) versus patients without evidence of metastasis (95%), similar to our cohort. The presence of tumor seeding in about half of patients and worse survival in bifocal GCTs supports the metastasis hypothesis [5,6].

Recent work by Zhang et al. has suggested that close review of clinical and imaging features can distinguish between “true” and “false” bifocal GCTs [12]. “True” bifocal tumors indicate a dual-primary, while the “false” bifocal is a metastasis from the pineal GCT to the floor of the III ventricle. They reported from an MRI analysis of 95 S-GCT in the literature that all neurohypophyseal germinomas exhibit thickening of the entire PS including the inferior pituitary stalk, which are the cardinal signs of “true” bifocal GCTs. “True” bifocal GCTs further demonstrated a larger extension of the tumor into the HNA, and a figure-eight or bottle-plug-shaped HNA. These tumors universally presented with DI. Zhang et al. concluded that primary germinomas that originated from the neurohypophysis grow toward the hypothalamus and have thickening of the entire PS including a thickened inferior PS. Conversely, in “false” bifocal GCTs, suprasellar lesions represent metastatic lesions to the III ventricle floor that grow toward the PS. On MR, they commonly exhibit a normal, irregular, or inverted cone shaped HNA and normal appearing PS [12], resembling Type IV tumors in our series.

#### 3.2.5. Implications of Bifocality for Irradiation

The natural course of bifocal germinomas is not significantly different from localized intracranial germinoma, which can be controlled with an extended focal radiotherapy field [6]. Whether or not bifocal GCTs represent metastatic lesions carries important implications for treatment, however. If bifocal lesions are considered metastases, empiric treatment with craniospinal irradiation (CSI) is indicated; if not, CSI should be reserved as a salvage therapy when local radiotherapy (RT) and chemotherapy fail [6]. A well-cited multicenter study by Calaminus et al. identified that patients with localized germinomas exhibited higher rates of treatment relapse with chemotherapy and focal RT alone versus CSI, although inclusion of the ventricles in the RT may make local therapy more effective [14]. Other studies have demonstrated high risk of late GCT recurrence with focal RT and chemotherapy alone, although CSI was found to be effective as a salvage therapy [15]. Zhang et al. recommended that since “false” bifocal GCTs represent metastasis, they warrant CSI, while “true” GCTs in the absence of other metastases can be treated with limited radiotherapy alone [12].

CSI in bifocal GCTs has been controversial, with some authors advocating initial CSI only in patients with disseminated disease, limiting RT fields until local RT fails [6]. More recent studies, however, have supported the bifocal metastatic hypothesis, pointing towards the high failure rate of local RT fields in localized disease and low toxicity of modern CSI to justify CSI in patients with bifocal disease [30,31,32]. Cuccia and Alderete defined bifocal GCT as a group of GCTs that lacked seeding between tumors or to distant sites and suggested that spinal radiotherapy was unnecessary [3]. Weksberg et al. divided bifocal GCTs into two groups: tumors without metastases (group 1) and tumors with metastases to other locations (group 2) [6]. They found that limited radiotherapy for group 1 tumors was associated with spinal failures. Another study by Phi et al. advocated that bifocal GCTs likely result from metastatic spread of suprasellar or pineal GCTs because nearly half of their patients with bifocal lesions presented with gross seeding or positive CSF cytology. Thus, they recommended CSI [5].

## 4. Materials and Methods

### 4.1. Data Acquisition

A consecutive review of all patients with intracranial GCTs managed at a tertiary academic pediatric neurosurgery service over a 30-year period (1988–2018) was performed. GCTs were defined as histologically confirmed tumors undergoing biopsy/resection or tumors that had both radiographic features and positive serum and/or cerebrospinal fluid (CSF) tumor markers indicative of a GCT. Project approval was obtained through the hospital institutional review board (study# 2005-12692).

Detailed clinical data on GCT patients were obtained from the data bank of Falk Brain Tumor Center at the Ann & Robert Lurie Children’s Hospital of Chicago, IL, USA. Demographics, including age at presentation and sex were documented for each patient, as well as symptoms, including hydrocephalus, abnormal endocrine function with particular attention to diabetes insipidus (DI), and visual deficits. Radiographic images, pathology reports, applied therapies, and treatment responses were also reviewed.

### 4.2. MR Classification

Pre-treatment MRI features for each patient were reviewed focusing on tumor location, extension and evidence of dissemination. All tumors involving the suprasellar location were included in this study. Tumors were classified into three groups: (1) solitary suprasellar lesions (S-GCT), (2) concurrent lesions in the suprasellar and pineal regions (bifocal GCTs), and (3) concurrent lesions in the suprasellar region and basal ganglia (BG-GCT). MR images were evaluated to analyze the characteristics of suprasellar lesions based on their location (III ventricle cavity, floor, suprasellar, parasellar and intrasellar) and appearance of pituitary stalk (PS) and pituitary gland (PG). The plane connecting the chiasm and mammillary body was considered to be the floor of the III ventricle. PS thickening was defined as 3.0 mm and greater in diameter or the presence of an abnormal nodule along the stalk [33].

Based on MR imaging findings, suprasellar lesions of each group (S-GCT, bifocal GCT, and BG-GCT) were classified based on the MR findings into five types as follows:

Type Ia: Globular anterior III ventricle lesion with identifiable PS and PG (Figure 1);
Type Ib: Globular anterior III ventricle lesion without identifiable PS but with identifiable PG (Figure 2);
Type II: Globular anterior III ventricle mass extending to the sella turcica (Figure 3);
Type III: Globular mass extending from the floor of III ventricle to sella turcica (Figure 4);
Type IV: Small laminar (L) or nodular (N) lesion at the floor of III ventricle extending to upper PS (Figure 5);
Type V: Limited to the PS (Figure 6).

### 4.3. Pathologic Classification

Metastases were defined as the presence of tumor in the ventricles or subarachnoid spaces outside the suprasellar or pineal regions on initial imaging or having positive CSF cytology for a GCT. MR findings before both RT/chemotherapy and afterward were recorded to evaluate treatment response and the appearance of the pituitary stalk and gland in each patient.

The pathology of each case was classified based upon the surgical specimen, CSF, and serum tumor markers. When the surgical biopsy specimen was not available for histological verification, the pathology was suspected based on tumor markers and responses to adjuvant therapy. GCTs were divided into two groups: germinoma and non-germinomatous GCTs (NGGCT). The presence of serum and CSF alpha fetoprotein (AFP) and beta HCG (β-HCG) were documented. AFP values of serum <10 ng/mL and CSF < 2 ng/mL were considered within the normal range. Greater AFP parameters were considered NGGCT with embryonal carcinoma or endodermal sinus tumor components. Serum and CSF β-HCG values above the reference range were considered abnormal. Tumors with β-HCG values >100 IU/L were considered to be NGGCT, while those above the reference age but below this value were suggestive of germinoma.

Adjuvant treatments (radiation and chemotherapy) were also documented for each patient. All patients were evaluated with MR during the chemotherapy and post-radiation periods. Progression-free survival (PFS) was defined as the time between clinical diagnosis and tumor growth on follow-up imaging, death, or the last clinical follow-up, whichever came first. Overall survival (OS) was defined as the time between clinical diagnosis and death, or, for survivors, the last clinical follow-up.

### 4.4. Statistical Analysis

Two-tailed Student’s t tests and analysis of variance (ANOVA) tests were performed to determine differences in numerical variables between the S-GCT, bifocal, and BG-GCT groups. Pearson’s chi-squared, and, if significant, Fisher’s exact tests were performed to calculate differences between categorical variables. Logrank (Mantel–Cox) tests were performed to calculate differences in overall and progression free survival. Statistically significant values were identified with a *P* less than 0.05, and confidence intervals defined at 95%. All statistics were performed using Statistical Analysis System (SAS) (SAS Institute, Inc., Cary, NC, USA), and Microsoft Excel (Microsoft, Redmond, WA, USA).

## 5. Conclusions

In this study, we explore a large cohort of patients with solitary suprasellar (S-GCT) and bifocal GCTs and demonstrate several important differences between the two groups. There is a striking difference in sexual distributions between solitary S-GCTs, which demonstrated a female predominance, and the other groups, which were almost exclusively male. As both solitary pineal GCTs and bifocal tumors both demonstrate similar male predominance, this suggests bifocal tumors are likely of pineal origin. A trend toward worse PFS and OS and higher rate of CSF seeding in the bifocal group is also notable, suggesting bifocal tumors represent either later-presenting disease or metastases.

We further introduce a novel MR classification that demonstrates a different radiologic appearance between isolated suprasellar tumors and suprasellar masses with additional lesions in the pineal region (bifocal tumors). S-GCTs almost uniformly present at the floor of the III ventricle and extend dorsally with or without an identifiable PS (Types Ia and Ib), ventrally (Type III) or in both directions (Type II). Small lesions limited to the anterior III ventricle floor or the PS are rare among S-GCTs. Conversely, the majority of bifocal GCTs show a small nodular or laminar lesion at the floor of the III ventricle extending to the upper PS (Type IV) with much larger pineal mass.

Taken together, a distinct radiographic appearance, marked male predominance, and much higher rate of metastases among the bifocal group suggests that a majority of bifocal GCTs represent metastases from the pineal to the suprasellar region. Exceptions may be present in cases with prolonged DI, the universal symptom for solitary GCTs, smaller pineal masses without hydrocephalus, and suprasellar lesions with BG-GCTs, which may represent either ventricular dissemination or peri-ependymal extension through the wall of the IIIrd ventricle.

The current study cannot definitively conclude whether bifocal GCTs represent concurrent dual primaries, subependymal extension from the pineal region to the suprasellar, or CSF-borne metastases. Radiation fields may be reduced in the former two, but the latter requires an extended radiation field. Localized S-GCTs are treated with WV RT, but in high risk groups such as metastatic disease or NGGCT, CSI is justified. For bifocal GCTs, WV is as effective as CSI if there are no signs of metastatic lesions. It may also be reasonable to have a higher level of concern for bifocal lesions that carry a distinctive MR appearance (Type IV) more suggestive of metastasis than those more common to S-GCTs (Types I-III). However, these recommendations should be tentative, and a larger prospective multicenter study is necessary to draw more definitive conclusions.

The primary limitation of this study is its small sample size and its retrospective nature. Although comparable to other studies of bifocal lesions in the literature [5,6,12], it is possible that many of the findings in this study that trended towards significance, including progression free and overall survival, may have been significant if additional data were available.

## Figures and Tables

**Table 1:** Magnetic resonance imaging types.

MR Imaging Type	S-GCT	Bifocal GCT	BG-GCT	Total
Type Ia	3 ^++^	2 ^+^	0	5
Type Ib	4 ^+^	2 ^+^	0	6
Type II	7 **	1	0	8
Type III	6 **	0	2 (L) ^++^	8
Type IV	1 (L) ^+^	11 (7 L ^++^; 4 N ^+++^)	0	12
Type V	1	0	2 ^++^	3
Total	22	16	4	

Type Ia: Globular anterior III ventricle mass lesion with identifiable pituitary stalk (PS) and pituitary gland (PG). Type Ib: Globular anterior III ventricle mass lesion without identifiable PS but with identifiable PG. Type II: Globular anterior III ventricle mass extending to the sella turcica. Type III: Globular mass extending from the floor of III ventricle to sella turcica. Type IV: Small laminar (L) or nodular (N) lesion at the floor extending to upper PS. Type V: limited to PS. Each asterisk (*) represents one patient with cavernous sinus involvement; each cross (^+^) represents one patient with ventricular metastases. Multiple asterisks or crosses represent multiple patients.

**Table 2:** Surgery.

Approach	S-GCT	Bifocal GCT	BG-GCT	Total
No biopsy	8 ^(2)^	5 ^(2)^	1	14
Endoscopic	2	9	0	11
Craniotomy	8	0	1	9
Transsphenoidal	4	1	0	5
Stereotactic	0	1	2	3
Total	22	16	4	42

The number in parenthesis (x) represents the number of patients undergoing second-look surgery after initial treatment with radiation or chemotherapy.

**Table 3:** Pathology.

Pathology	S-GCT	Bifocal GCT	BG-GCT	Total
Germinoma (diagnosed)	10	11	3	24
Germinoma (suspected)	5	3	1	9
NGGCT	7	2		9
Alpha fetoprotein (AFP)	3	2		
β-HCG >100 IU/L	3			
AFP + β-HCG >100 IU/L	1			
Total	22	16	4	

Four of the seven suprasellar non-germinomatous germ cell tumors (NGGCTs) had a mature teratoma component. Two mature teratomas were identified surgically before adjuvant treatment, and two during the second look surgery. Of the two bifocal NGGCTs, the second look surgery showed no viable tumors.

**Table 4:** Radiation therapy field and intraventricular metastases.

Radiation Therapy	S-GCT		Bifocal GCT		BG-GCT	Total
	Germinoma	NGGCT	Germinoma	NGGCT	Germinoma	
Whole ventricle	9 ^(2)^		8 ^(1)^			17 ^(3)^
CSI	1 ^(1)^	6	7 ^(5)^	1 ^(1)^	4 ^(3)^	19 ^(10)^
Involved field	4	1 ^(1)^				5 ^(1)^
Whole brain	1					1
Total	15 ^(3)^	7 ^(1)^	15 ^(6)^	1 ^(1)^	4 ^(3)^	42 ^(14)^

The number in parenthesis (x) represents the number of patients with intraventricular metastases before therapy. CSI: craniospinal irradiation.

**Table 5:** Radiation therapy field and recurrences.

Radiation Therapy	S-GCT		Bifocal GCT		BG-GCT	Total
	Germinoma	NGGCT	Germinoma	NGGCT	Germinoma	
Whole ventricle	9 ^(1)^		8 ^(2)^			17 ^(3)^
CSI	1	6 ^(2)^	6 ^(1)^	2 ^(2)^	4	19 ^(5)^
Involved field	4	1 ^(1)^				5 ^(1)^
Whole brain	1					1
Total	15 ^(1)^	7 ^(3)^	14 ^(3)^	2 ^(2)^	4	42 ^(9)^

The number in parenthesis (x) represents the number of patients with recurrence.
